# Pharmacotherapy for mild hypertension

**DOI:** 10.1590/S1516-31802012000600012

**Published:** 2013-01-18

**Authors:** Diana Diao, James M. Wright, David K. Cundiff, Francois Gueyffier

## Abstract

**BACKGROUND:**

People with no previous cardiovascular events or cardiovascular disease represent a primary prevention population. The benefits and harms of treating mild hypertension in primary prevention patients are not known at present. This review examines the existing randomized controlled trial (RCT) evidence.

**OBJECTIVE:**

Primary objective: To quantify the effects of antihypertensive drug therapy on mortality and morbidity in adults with mild hypertension (systolic blood pressure (BP) 140-159 mmHg and/or diastolic BP 90-99 mmHg) and without cardiovascular disease.

**METHODS:**

*Search:* We searched CENTRAL (2011, Issue 1), MEDLINE (1948 to May 2011), EMBASE (1980 to May 2011) and reference lists of articles. The Cochrane Database of Systematic Reviews and the Database of Abstracts of Reviews of Effectiveness (DARE) were searched for previous reviews and meta-analyses of anti-hypertensive drug treatment compared to placebo or no treatment trials up until the end of 2011.

*Selection criteria:* RCTs of at least 1 year duration.

*Data collection and analysis:* The outcomes assessed were mortality, stroke, coronary heart disease (CHD), total cardiovascular events (CVS), and withdrawals due to adverse effects.

**MAIN RESULTS:**

Of 11 RCTs identified 4 were included in this review, with 8,912 participants. Treatment for 4 to 5 years with antihypertensive drugs as compared to placebo did not reduce total mortality (RR 0.85, 95% CI 0.63, 1.15). In 7,080 participants treatment with antihypertensive drugs as compared to placebo did not reduce coronary heart disease (RR 1.12, 95% CI 0.80, 1.57), stroke (RR 0.51, 95% CI 0.24, 1.08), or total cardiovascular events (RR 0.97, 95% CI 0.72, 1.32). Withdrawals due to adverse effects were increased by drug therapy (RR 4.80, 95% CI 4.14, 5.57), ARR 9%.

**AUTHORS’ CONCLUSIONS:**

Antihypertensive drugs used in the treatment of adults (primary prevention) with mild hypertension (systolic BP 140-159 mmHg and/or diastolic BP 90-99 mmHg) have not been shown to reduce mortality or morbidity in RCTs. Treatment caused 9% of patients to discontinue treatment due to adverse effects. More RCTs are needed in this prevalent population to know whether the benefits of treatment exceed the harms.

**This is the abstract of a cochrane review published in the Cochrane Database of Systematic Reviews (CDSR) 2012, issue 8, DOI:** 10.1002/14651858.CD006742.pub8 (http://onlinelibrary.wiley.com/doi/10.1002/14651858.CD006742.pub2/abstract). For full citation and authors details see reference 1.

**The full text is freely available from:** http://cochrane.bvsalud.org/cochrane/show.php?db=reviews&mfn=4030&id=CD006742&lang=pt&dblang=&lib=COC&print=yes#
